# Preliminary Results of the First 50 Patients Undergoing Sclerotherapy for II-Degree Hemorrhoidal Disease Using an Automated Device

**DOI:** 10.3389/fsurg.2022.882030

**Published:** 2022-04-14

**Authors:** Marta Goglia, Casimiro Nigro, Paolo Aurello, Elia Diaco, Mario Trompetto, Gaetano Gallo

**Affiliations:** ^1^Department of Surgery, Sant'Andrea Hospital, Sapienza University of Rome, Rome, Italy; ^2^Department of General Surgery, University of Rome “Tor Vergata, ” Rome, Italy; ^3^Minerva Surgical Service, Catanzaro, Italy; ^4^Department of Colorectal Surgery, S. Rita Clinic, Vercelli, Italy; ^5^Unit of General Surgery and Surgical Oncology, Department of Medicine, Surgery, and Neurosciences, University of Siena, Siena, Italy

**Keywords:** hemorrhoidal disease, sclerotherapy, 3% polidocanol foam, bleeding haemorrhoids, symptomatic haemorrhoids

## Abstract

**Background:**

Sclerotherapy is defined as the injection of sclerosant agents causing fibrosis and scarring of the surrounding tissue. It is currently employed for the treatment of I-III degree hemorrhoidal disease (HD). The aim of this study is to investigate the use of a new automated device for the injection of 3% polidocanol foam.

**Methods:**

This is an observational study including 50 patients who underwent a sclerotherapy procedure with 3% polidocanol foam for II-degree HD according to Goligher classification. Patients were evaluated through validated scores [Giamundo score, Hemorrhoidal Disease Symptom Score (HDSS), Short Health Scale (SHS-HD) and Vaizey score]. Follow-up was conducted until 3 months from the procedure.

**Results:**

Complete resolution of bleeding was achieved in 72% and 78% of patients, respectively, at 1 week and after 3 months from the procedure. Forty eight percent of patients were symptom free after the last follow-up visit (HDSS = 0). No major surgical complications were reported. Three patients out of 36 successfully treated, recurred, and needed a second sclerotherapy injection, which was successful in 2 of them.

**Conclusion:**

These preliminary results of 3% polidocanol foam injection on 50 patients suggest the efficacy and reproducibility of the technique with this new device in the short-term follow-up.

## Background

Hemorrhoidal disease (HD) represents a common clinical condition in the Western world among the adult population (1). During the last years, its impact on the quality of life and on the patients' daily activities has been largely discussed in the scientific literature due to its high incidence, multifactorial aetiology, and the absence of a strict consensus regarding diagnosis and therapeutic assessment (2).

Historically, HD has been classified according to the Goligher classification, even though this last one is, today, considered incomplete, and it requires other methods of grouping (3). However, the choice of treatment today, going from conservative to surgical ones, is guided on the severity of disease presentation and related symptomatology.

Several studies demonstrated successful results after sclerotherapy in terms of remission of the symptomatology, complication rates, and cost-effectiveness in those patients who presented with I-III degree of HD (4, 5). Sclerotherapy is defined as the injection of sclerosing agents at the apex of the internal hemorrhoidal complex, above the dentate line, leading to scarring, fibrosis, and fixation of the hemorrhoids (2). The aim of this study is to investigate the efficacy, safety, and reproducibility of a new technique by the means of a newly introduced automated device (Varixio © VB Devices, Barcelona, Spain) for the injection of a 3% polidocanol foam.

## Methods

This is an observational study including 50 patients who underwent an elective sclerotherapy procedure for II-degree HD according to Goligher classification. All patients failed conservative treatment.

Results were reported according to the Strengthening the Reporting of Observational Studies in Epidemiology (STROBE) statement for cohort studies (6).

The patients were enrolled between August and October 2021; their follow-up continued until January 2022. All the patients underwent elective surgical treatment on an outpatient basis by an experienced colorectal surgeon.

All the patients who underwent treatment with sclerotherapy, reaching at least 3 months of follow-up, were included in the analysis. Indeed, during the study period, other 19 procedures were performed without achieving the required follow-up.

The second sclerotherapy session was performed at least 4 weeks after the first injection. The same criterion was used for any subsequent repetitions.

Surgery was performed only after a complete work-up including the patient's history, stratification of the patient in degrees of severity by means of validated scores, and physical proctological examination through anoscopy.

Colonoscopy was performed in suspicious cases for rolling out other colorectal disorders.

**Figure 1:**
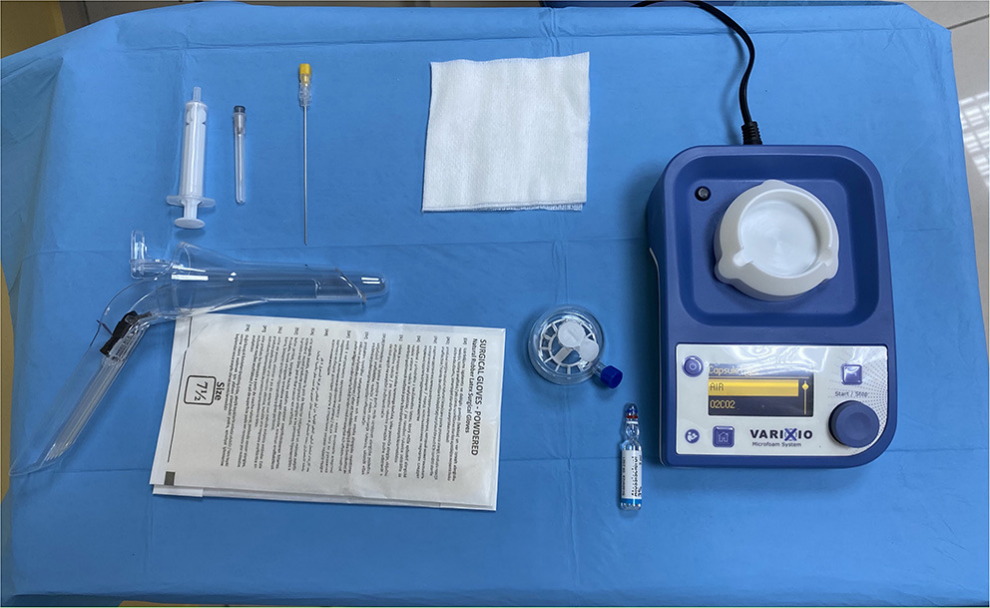
The equipment needed to perform the procedure: an automated device, open-ended anoscope, a 20 Gauge needle, a 5-ml syringe, and polidocanol 3% liquid.

The procedure was performed in the Sims position on an outpatient setting without any sedation or local anaesthesia as previously described (7, 8). It consisted of the injection of a 3% polidocanol foam using a new automated device (Varixio © VB Devices, Barcelona, Spain) (Figure 1). This device allows a continuous foam injection reducing, at minimum, the human error. It is composed of a dome-shaped capsule, with a luer lock both for injection and removal of the sclerosing agent and a magnetic stirrer on top. The foam demonstrated to have a stable consistency and a higher 1.5-x/2-x half life with respect to the liquid sclerosant used in the Tessari method. Moreover, the device allows the use of different percentages of composition, maintaining a liquid/gas ratio between 1:5 and 1:7. It constantly re-emulsifies the foam during the whole duration of the procedure and reduces the human error so drastically to be now considered an operator-independent technique. The emulsified foam has a bubble diameter (μm) of 116 ± 24 with a half-life of 5.2 ± 0.6 min (9). A total of 2 ml of 3% polidocanol foam was used for each of the three classical piles (3, 7, and 11 o'clock).

After the procedure, the patients were asked to walk and were discharged 20 min later, after a safety check. Stool softeners and flebotonics were administered in the post-operative period.

Time of procedure was considered as a baseline (T0) and after that, the patients underwent follow-up visits, which consisted of external clinical evaluation after 1 week (T1), and a complete proctological evaluation, including digital rectal examination and anoscopy after 4 weeks (T2) and 3 months (T3). The patients were evaluated by validated scores. Giamundo score was utilised for the evaluation of bleeding at the baseline, and all follow-up visits (0 = absence of bleeding, 1 = <1 episode per month, 2 = 1 episode per week, 3 = 1–3 episodes per week and 4 = 4 or more episodes per week) (10).

Symptom severity and quality of life were assessed using a five-item questionnaire: the Hemorrhoidal Disease Symptom Score (HDSS). This score evaluates pain, itching, bleeding, soiling, and prolapse on a 5-point scale (0 = never, 1 = less than one time a month, 2 = less than one time a week, 3 = 1–6 days per week, 4 every day or always). Another used score was the Short Health Scale for HD (SHS-HD) score, including 4 questions with a 7-point Likert scale for each question minimum score = 0, 7 = maximum score = 0 at T0, and T3 (11). Anal continence was evaluated through the Vaizey incontinence score (minimum score = 0, perfect continence/maximum score = 24, totally incontinent) at T0, T2, and T3 (12).

Visual analogue scale (VAS) score was used to evaluate peri-procedural pain (VAS) score (minimum score = 0, maximum score = 10).

The primary outcome was defined as the complete resolution of bleeding episodes 1 week after the procedure based on the Giamundo bleeding score.

Recurrences were defined as the new onset of bleeding after T1 in the successfully treated patients, always based on Giamundo score assessment, from a bleeding score of 0 to at least 2 at any time point between T2 and T3.

### Eligibility Criteria

Patients aged between 18 and 75 years with symptomatic II-degree HD according to the Goligher classification were considered eligible for the present study.

The patients with a history of cardiac disease, blood disorders, gastrointestinal tract oncological or inflammatory disorders, other proctological diseases, previous anal surgical procedures, recurrency of the pathology after sclerotherapy or rubber band ligation in the last 12 months, pregnancy or lactation, infectious disease, or previous pelvic radiotherapy were excluded. The inability to return for postoperative control visits was also considered an exclusion criterion.

### Safety

Safety and toxicity of the procedure were investigated by reporting the adverse events (AE) after foam injection and using the WHO toxicity scale, respectively (13). AEs were reported according to the probability of occurrence as none, remote, possible, probable, or not assessable.

### Statistical Analysis

Categorical variables were analysed and reported as counts and percentages, and as the mean ± SD (range) for continuous normally distributed variables, whereas ordinal categorical variables, and continuous not normally distributed variables were reported as median [interquartile range (IQR)]. The chi-square test was used for cross tabulations. The results associated with a *p* < 0.05 were considered statistically significant.

## Results

Most of the population enrolled in the study was male, consisting of 31 patients out of 50 (62%), whereas the rest, the other 19 patients out of 50 (38%), were females. The mean age of the population was 48.3 ± 17.2 (range: 18–75) years. The mean operation time was 5.5 ± 1.7 (range: 3–10) min (Table 1).

**Table 1:** Patient characteristics and procedural results.

Male (*N*°, %)	31/50 (62%)
Mean age (years)	48.3 ± 17.2 (range: 18–75)
Mean operation time (minutes)	5.5 ± 1.7 (3–10)
Post-operative Pain (VAS > 0)	3/50 (6%) 0 (0–0)
Success Rate after 1 week (T1)	36/50 (72%)
Overall Success Rate (T5)	39/183 (78%)
Recurrence	3/36 (8.3%)
Adverse Events	None

At the baseline (T0), Giamundo score had a mean of 3.18 ± 0.63 (range: 2–4), meaning that the majority of the patients, graded II according to the Goligher classification of HD, referred 1–3 episodes of bleeding per week before treatment. After 1 week, the complete resolution of bleeding was achieved in 36 out of 50 patients (72%), whereas only 12 patients reported an episode of bleeding (Giamundo score = 1) with a mean of.32 ± 0.55 (range: 0–2) with respect to the total population.

Giamundo score maintained stable values even at the second follow-up visit after 1 month from the procedure (T2). It reported a mean value of 0.56 ± 0.93 (range: 0–3) with 17 patients referring at least one bleeding episode in the last month.

However, the analysis at the third and last follow-up visits at 3 months from the procedure confirmed values stable in time for the Giamundo score with only 11 patients reporting a score equal or higher than 1 with a mean value of 0.3 ± 0.68 (range: 0–3) with an overall success rate of 78% (39/50). The differences of the Giamundo score were highly statistically significant (*p* < 0.0001) (Table 2).

**Table 2:** Differences among the mean of the Giamundo score at baseline (T0) and follow-up visits.

**Mean**	**T0**	**T1**	**T2**	**T3**	***P*-value**
Giamundo score	3.18	0.32	0.56	0.3	<0.0001

In 2 patients (4%), there was worsening of the Giamundo score at T2, from 1 to 3. For these patients, a second sclerotherapy session has been successfully performed. One patient got worse at T3, from 1 to 3, and a second sclerotherapy session has been planned but not included in the results.

About 3 out of 36 (8.3%) successfully treated patients recurred according to the primary outcome, and, after the second sclerotherapy session, one patient became successful, one patient improved from 3 to 1, and one patient failed (remaining at Value 3 from preoperative to postoperative examination).

Vaizey score at the baseline was considered completely negative with a median value of 0 (IQR: 0–1). It demonstrated to maintain the same results (median of 0, IQR: 0–0) at a T2 follow-up visit with a statistically significant *p* value (*p* < 0.021).

On the other side, median HDSS was 11 preoperatively (IQR: 9–12), considerably improving at the T3 follow-up to a median value of 1.5 (IQR: 0–3); the difference was statistically significant (*p* < 0.0001). Forty eight percent of patients were symptom free (HDSS = 0) after the last follow-up visit.

Similarly, improvement of the SHS-HD was statistically significant (*p* < 0.0001) from a median value of 16 (IQR: 14–18) to a reported value of 0 (IQR: 0–5) (Table 3).

**Table 3:** Differences among the median values of Hemorrhoidal Disease Symptom Score (HDSS) and Short Health Scale (SHS) between baseline (T0) and 3 month follow up (T3).

**Median**	**T0**	**T3**	***P*-value**
HDSS	11	1.5	<0.0001
SHS	16	0	<0.0001

Only three patients referred to peri-procedural pain on the VAS score (<3), with a median value of 0 (IQR 0–0). No other intraoperative complication was registered.

## Discussion

The results of this preliminary analysis on the treatment of HD with polidocanol 3% foam demonstrated the safety as well as the efficacy of the procedure performed through a new automated device (Varixio © VB Devices, Barcelona, Spain). Indeed, neither intraoperative nor postoperative complications occurred. Our results reported a great improvement of bleeding symptoms for the majority of the population. Over 78% of the patients maintained a therapeutical success at 3-month follow-up, and almost half of the entire population was symptom free at the last follow-up visit based on the HDSS score. The severe standardisation of the procedure as well as the use of validated scores allowed to objectify the results, avoiding a difficult interpretation of the latter.

The patients who suffer from HD generally bear a great psychological discomfort because of recurrent presentation of symptomatology, not responding to medical therapy, feeling of shame, and fear of surgery; therefore, there is a high number of patients that seek the help of the physician when the disease is already advanced in its severity. Nevertheless, those patients who present to medical attention with I-II and, sometimes, III-degree HD might be successfully treated on the outpatient clinics, with minimally invasive procedures like sclerotherapy or rubber band ligation. The spread of this less-invasive procedure may lead to the general population to consult their general practitioner earlier.

Sclerotherapy is currently recognised as an efficacious method of treatment in I and II-degree HD through the injection of sclerosing materials with benefits for the patients in terms of recovery and minimal discomfort that allow normal continuation of daily-life activities. Moreover, this technique is safe, cheap, and easy to run, permitting its application also in tough cases and in III-degree HD, who failed conservative treatment (2). In fact, in cases in which the patient has many comorbidities and is not fit for anaesthesia, or is waiting for a more invasive therapy, he/she may benefit from a damage-control assessment. For example, in case of severe acute anal bleeding, a bridge to surgery through sclerotherapy might be the most appropriate choice of treatment (14).

Recent studies have demonstrated the higher efficacy of foam injection with respect to the one involving liquid sclerosing agent (5). This new device (Varixio © VB Devices, Barcelona, Spain) allows the injection of a continuously re-emulsified foam and reduce at minimum the success variability related to the different operators. The present study is the first one reporting the procedural results after 3% polidocanol foam injection using an automated device.

Over the last few years, there has been an increasing attention and appreciation of sclerotherapy with 3% polidocanol foam (4, 15, 16).

Lobascio et al. (4) published similar results of the therapeutical success rate in a limited population with a longer follow-up of 12 months. The authors reported 78.8% of success after a single-ST session with around 20% of recurrences in the first 6 months treated with a second ST injection (with a success rate of 86%) or with mucopexy.

Salgueiro et al. (15) published the first randomised trial regarding the comparison between rubber band ligation and sclerotherapy with 3% polidocanol foam injection. The authors registered a rate of success of sclerotherapy similar to ours, even though the two studies are not comparable due to the different study designs and primary endpoints. They reported a higher complete success rate in the sclerotherapy group with respect to the rubber band ligation, particularly in the 88.3 and 66.7% (*p* = 0.009) of the patients, respectively. Moreover, the recurrence rate (16.1 vs. 41.1%; *p* = 0.004), and complications (10 vs. 30%; *p* = 0.01) were inferior in case of sclerotherapy.

The conclusions of both studies agree with ours. In addition to this confirmation, the new device that we utilised during this study contributes to the efficacy and reproducibility of the procedure with technical improvements related specifically to the instrument. Indeed, the variability of foam consistency is completely abolished, thanks to the continuous emulsification of the foam during the procedure, as already explained. The real strength of this method is that the whole operation is made faster and reproducible by every surgeon, limiting human error to a minimum. However, this technique deserves further studies in a wider sample of patients to better evaluate the efficacy and the long-term results. Limitations of the present study are the retrospective analysis of the data, even though the enrolment has been prospective, the limited sample of the patients, and the limited duration of follow-up until 3 months.

## Conclusion

Sclerotherapy is a valid therapeutical option in case of bleeding HD.

Preliminary results of polidocanol injection with this new device on 50 patients suggest the efficacy of the technique in the short-term follow-up. This technique is safe and repeatable, useful in case of a bridge to surgery and in damage control emergency procedures. However, other investigations in a broader population sample and with a longer follow-up are needed.

## Data Availability Statement

The raw data supporting the conclusions of this article will be made available by the authors, without undue reservation.

## Ethics Statement

The studies involving human participants were reviewed and approved by Regione Calabria–Comitato Etico Sezione Area Centro. The patients/participants provided their written informed consent to participate in this study.

## Author Contributions

MG and GG: substantial contributions to the conception and design of the work, acquisition, analysis, interpretation of data for the work, drafting the work, revising it critically for important intellectual content, final approval of the version to be published, and agreement to be accountable for all aspects of the work in ensuring that questions related to the accuracy and integrity of any part of the work are appropriately investigated and resolved. CN, PA, ED, and MT: substantial contributions to the conception and design of the work, acquisition, analysis, interpretation of data for the work, and agreement to be accountable for all aspects of the work in ensuring that questions related to the accuracy and integrity of any part of the work are appropriately investigated and resolved. All the authors contributed to the article and approved the submitted version.

## Conflict of Interest

The authors declare that the research was conducted in the absence of any commercial or financial relationships that could be construed as a potential conflict of interest.

## Publisher's Note

All claims expressed in this article are solely those of the authors and do not necessarily represent those of their affiliated organizations, or those of the publisher, the editors and the reviewers. Any product that may be evaluated in this article, or claim that may be made by its manufacturer, is not guaranteed or endorsed by the publisher.

## References

[1] Gallo G Sacco R Sammarco G. Epidemiology of hemorrhoidal disease. In: Ratto C, Parello A, Litta F, editors. Hemorrhoids Coloproctology. Springer, Cham (2018).

[2] Gallo G Martellucci J Sturiale A Clerico G Milito G Marino F. Consensus statement of the Italian society of colorectal surgery (SICCR): management and treatment of hemorrhoidal disease. Tech Coloproctol. (2020) 24:145–64. 10.1007/s10151-020-02149-1 31993837 PMC7005095

[3] Dekker L Han-Geurts IJM Grossi U Gallo G Veldkamp R. Is the Goligher classification a valid tool in clinical practice and research for hemorrhoidal disease? Tech Coloproctol. (2022). 10.1007/s10151-022-02591-3 35141793 PMC9018630

[4] Lobascio P Laforgia R Novelli E Perrone F Di Salvo M Pezzolla A. Short-term results of sclerotherapy with 3% polidocanol foam for symptomatic second- and third-degree hemorrhoidal disease. J Invest Surg. (2021) 34:1059–65. 10.1080/08941939.2020.1745964 32290709

[5] Moser KH Mosch C Walgenbach M Bussen DG Kirsch J Joos AK. Efficacy and safety of sclerotherapy with polidocanol foam in comparison with fluid sclerosant in the treatment of first-grade haemorrhoidal disease: a randomised, controlled, single-blind, multicentre trial. Int J Colorectal Dis. (2013) 28:1439–47. 10.1007/s00384-013-1729-2 23775099

[6] Von Elm E Altman DG Egger M Pocock SJ Gøtzsche PC Vandenbroucke JP Initiative STROBE. The strengthening the reporting of observational studies in epidemiology (STROBE) statement: guidelines for reporting observational studies. Int J Surg. (2014) 12:1495–9. 10.1016/j.ijsu.2014.07.013 25046131

[7] Gallo G Trompetto M Diaco E. The use of a new automated device for the sclerosing treatment of haemorrhoidal disease - a video-vignette. Colorectal Dis. (2021). 10.1111/codi.15992 34796600

[8] Lobascio P Minafra M Laforgia R Giove C Trompetto M Gallo G. The use of sclerotherapy with polidocanol foam in the treatment of second-degree haemorrhoidal disease - a video vignette. Colorectal Dis. (2019) 21:244–5. 10.1111/codi.14498 30485654

[9] Roche E Pons R Roche O Puig A. A new automated system for the preparation of sclerosant foam: a study of the physical characteristics produced and the device settings required. Phlebology. (2020) 35:724–33. 10.1177/0268355520937615 32635818

[10] Giamundo P Braini A Calabro' G Crea N De Nardi P Fabiano F. Doppler-guided hemorrhoidal dearterialization with laser (HeLP): a prospective analysis of data from a multicenter trial. Tech Coloproctol. (2018) 22:635–43. 10.1007/s10151-018-1839-5 30159627

[11] Rørvik HD Styr K Ilum L McKinstry GL Dragesund T Campos AH. Hemorrhoidal disease symptom score and short health scaleHD: new tools to evaluate symptoms and health-related quality of life in hemorrhoidal disease. Dis Colon Rectum. (2019) 62:333–42 10.1097/DCR.0000000000001234 30451751

[12] Vaizey CJ Carapeti E Cahill JA Kamm MA. Prospective comparison of faecal incontinence grading systems. Gut. (1999) 44:77–80. 10.1136/gut.44.1.77 9862829 PMC1760067

[13] Miller AB Hoogstraten B Staquet M Winkler A. Reporting results of cancer treatment. Cancer. (1981) 47:207–14. 10.1002/1097-0142(19810101)47:1<207::aid-cncr2820470134>3.0.co;2-6 7459811

[14] Gallo G Ronconi M Trompetto M. Sclerotherapy with 3% polidocanol foam: revolutionizing outpatient treatment in patients with haemorrhoidal disease. Updates Surg. (2021) 73:2029–30. 10.1007/s13304-021-01008-4 33660166 PMC7927757

[15] Salgueiro P Garrido M Gaio R Pedroto I Castro-Poças F. polidocanol foam sclerotherapy versus rubber band ligation in hemorrhoidal disease grades I/II/III: randomized trial. Dis Colon Rectum. (2021). 10.1097/DCR.0000000000002117 34840294

[16] Gallo G Pietroletti R Novelli E Sturiale A Tutino R Lobascio P. A multicentre, open-label, single-arm phase II trial of the efficacy and safety of sclerotherapy using 3% polidocanol foam to treat second-degree haemorrhoidal disease (SCLEROFOAM). Tech Coloproctol. (2022). 10.1007/s10151-022-02609-w 35334004 PMC8949823

